# Supplementary material for: Exposure of Pseudomonas aeruginosa to Cinnamaldehyde Selects Multidrug Resistant Mutants
Source: Antibiotics (Basel). 2022 Dec 10;11(12):1790. doi: 10.3390/antibiotics11121790 (PMC9774640; doi:10.3390/antibiotics11121790)
Supplement: Supplementary file 1 [file antibiotics-11-01790-s001.zip › antibiotics-2075011-supplementary.pdf]

Figure S1. Phylogenetic tree of CNA-adaptation mutants derived from PA14. Mutants CNA-A1 to A3 were selected on a fixed concentration (Method A) of CNA (900 µg/mL) for 10 days. Mutants CNA-B1 to B7 were selected on increasing CNA concentrations (Method B), after 5 days (CNA-B1, B2 and B3) or 10 days of exposure (CNA-B4, B5, B6 and B7). Posterior probability, representing the fraction of trees where this split is observed, is indicated at branches.

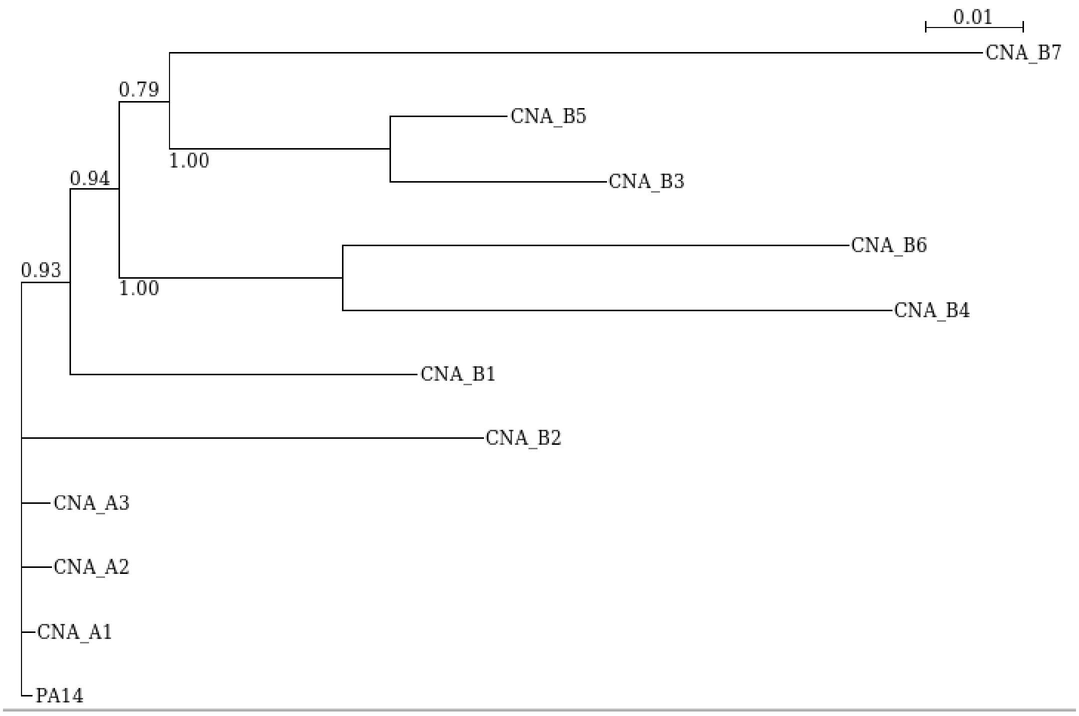

Figure S2. Structural alignment of NalC N-terminal region (1-108 over 209 amino acids) with representative TetR family proteins of known structures. TetR from *Escherichia coli* (UniProt code P0ACT4; PDB code 1QPI), TetR from of *Mesorhizobium japonicum* (UniProt code Q98D70; PDB code 3BHQ), DesT from *Pseudomonas aeruginosa* (UniProt code Q9HUS3; PDB code 3LSP), LuxR *from Vibrio cholerae* (UniProt code B4X9Q4; PDB code 7AMN) and MtrR from *Neisseria gonorrhea* (UniProt code P39897; PDB code 7JPN). Alignment was made with ESPript program [1]. Predicted secondary structure of NalC model is shown above the aligned sequences (α = helix α). Conserved residues are indicated in red and consensus residues are indicated in yellow. Mutation T24P in NalC is indicated with an arrow. NalC exhibits 24.4%, 20.9%, 23.4%, 20.7% and 20% of sequence identity with TetR *M. japonicum*, TetR E. *coli*, DesT, LuxR and MtrR, respectively (Clustal Omega; [2]). DNA binding domain, overlapping helix α1-3 (from residue 27 to 48), is represented as a blue arrow.

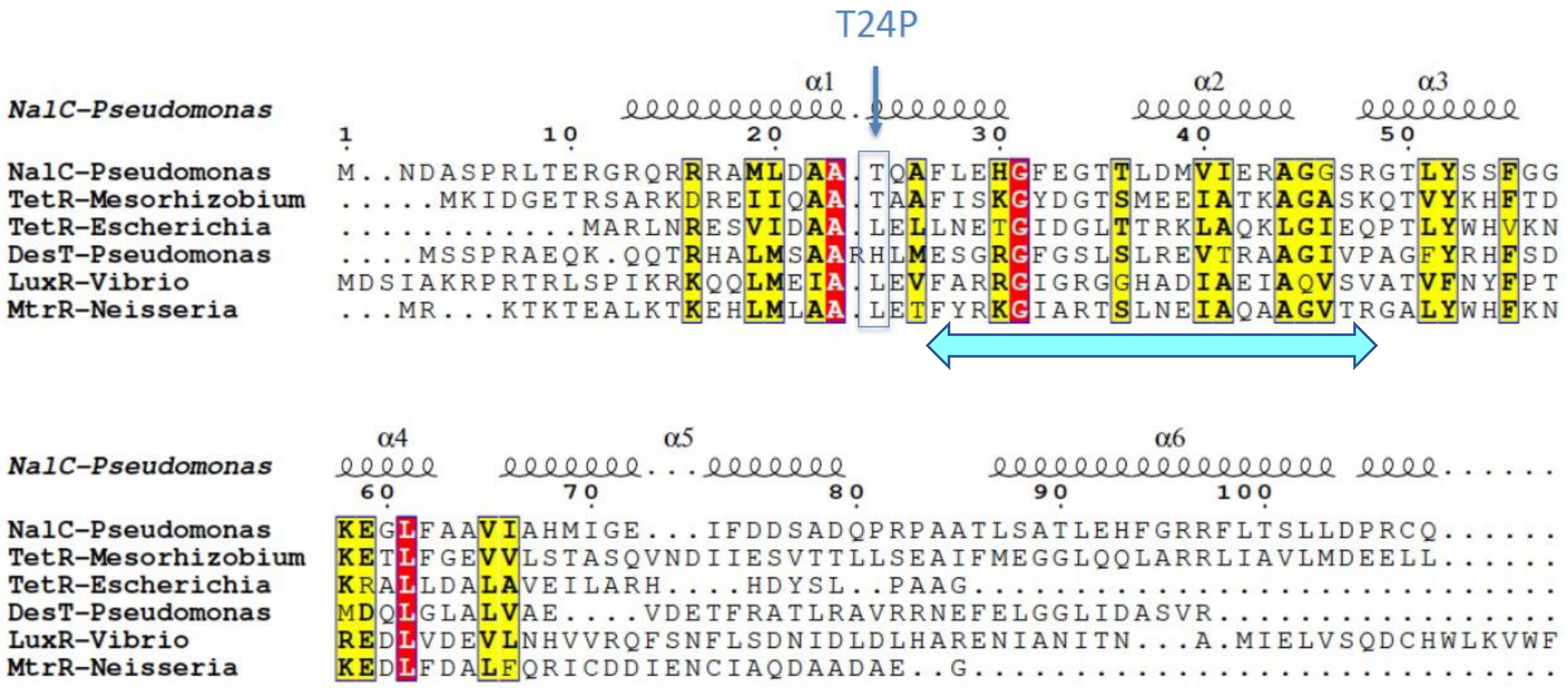

Figure S3. NalC model (homodimer) with a cognate DNA. The model of NalC homodimer in complex with DNA was generated based on the superposition of NalC model with TetR-DNA crystal structure from *E. coli* (PDB code 1QIN) using PyMOL program (Molecular Graphics System, Version 2.0 Schrödinger, LLC). One of NalC monomers is represented in rainbow colors (from N-terminal blue to C-terminal red). The α-helix numbering nomenclature is indicated according to TetR structure. Hydrogen bonds are shown as yellow dashed-lines. The NalC putative substrate-binding site was defined according to the superposition of NalC with TetR tetracycline binding pocket (indicated in pink). Because of the $T_{24}P$ substitution, the hydrogen bond with R106 of helix α7 and intra-chain hydrogen bond of helix α1 is expected to be disrupted.

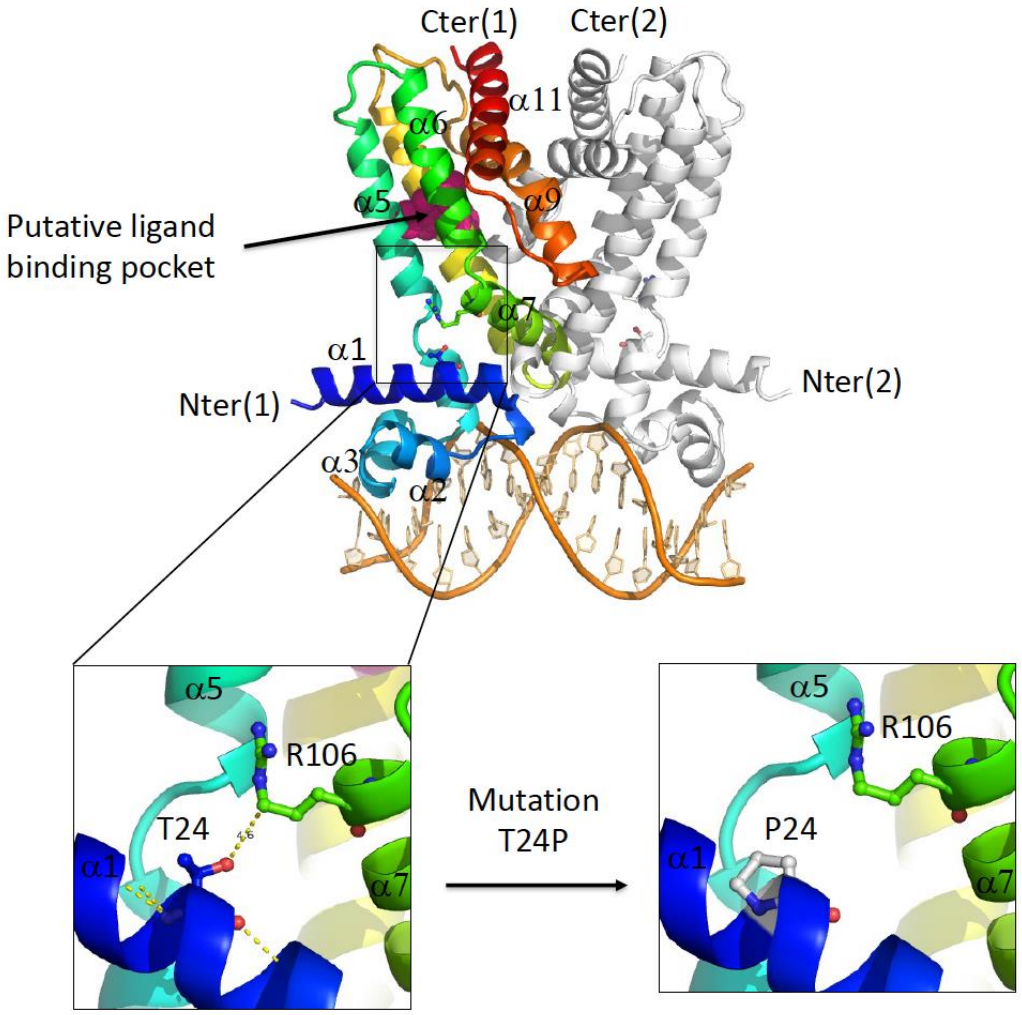

Figure S4. CNA degradation and conversion into cinnamic alcohol (CNOH) in PA14, A-type and B-type CNA-adaptation mutants. CNA and CNOH were assayed from culture supernatant by thin-layer chromatography 1h after addition of 1 mg/mL CNA.

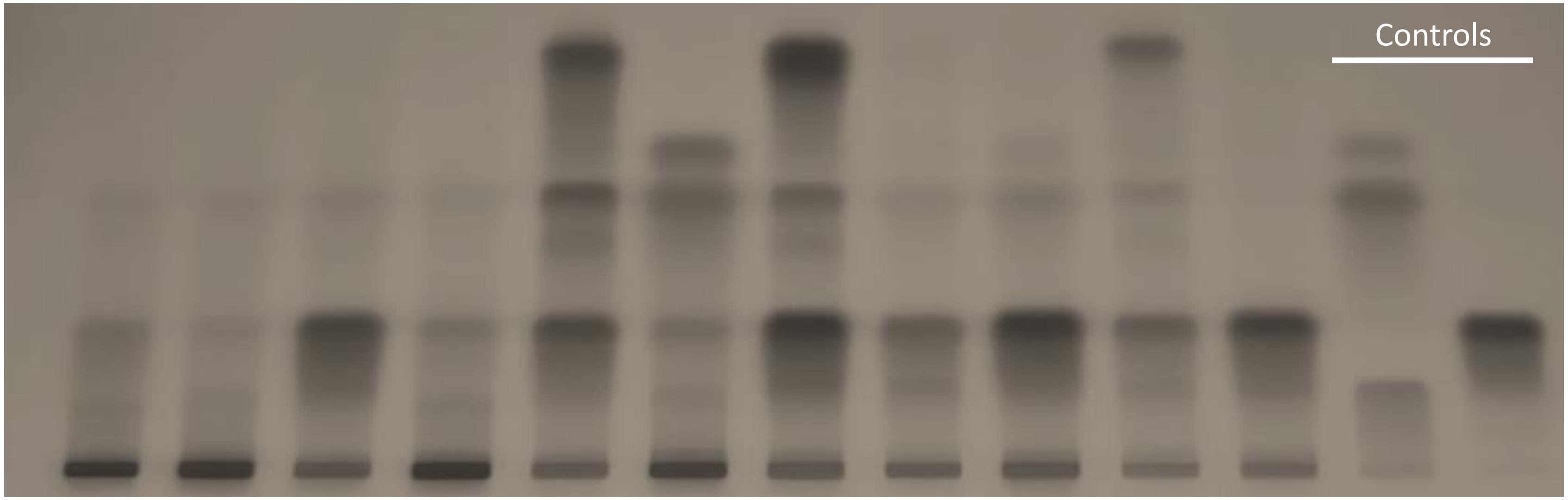

Strains	PA14	CNA-A1	CNA-A2	CNA-A3	CNA-B1	CNA-B2	CNA-B3	CNA-B4	CNA-B5	CNA-B6	CNA-B7	**CNA**	**CNOH**
**CNA MIC (µg/ mL)**	**700**	**900**	**900**	**800**	**1000**	**800**	**1000**	**1100**	**1000**	**1100**	**1100**		

Table S1. Susceptibility of CF strains P6-1 and P6-21 and their derivatives (-m1 to -m62) to antibiotics.

	Inhibition zone around antibiotic (mm)*											
	AKN	GME	TMN	MEM	IPM	CAZ	FEP	CIP	ATM	PIR	TIC	COL
**P6-1**	**26**	**26**	**26**	**35**	**27**	**27**	**28**	**34**	**28**	**25**	**24**	**15**
P6-1-m1	25	27	26	34	28	28	27	34	29	24	25	15
P6-1-m2	13	22	21	14	10	27	24	15	27	18	25	19
P6-1-m3	23	27	26	35	26	30	26	35	27	24	23	14
P6-1-m4	23	25	24	32	26	27	28	35	27	23	24	13
P6-1-m5	25	27	25	35	27	30	28	36	29	24	23	15
P6-1-m6	25	28	26	36	28	31	30	33	30	25	22	15
**P6-21**	**16**	**20**	**21**	**15**	**11**	**22**	**19**	**17**	**22**	**11**	**20**	**19**
P6-21-m1	18	21	23	17	11	29	23	17	29	20	24	20
P6-21-m2	18	23	24	20	12	32	26	18	32	24	27	20
P6-21-m3	17	23	25	20	12	31	24	18	30	23	29	21
P6-21-m4	17	23	24	16	11	27	22	17	30	18	28	20
P6-21-m5	18	26	26	26	10	38	32	15	40	26	34	21
P6-21-m6	20	25	26	15	10	30	24	15	31	20	26	20
P6-21-m7	18	25	26	20	10	31	26	20	31	21	29	21
P6-21-m8	16	22	23	20	15	31	28	18	17	24	34	19
P6-21-m9	18	24	25	14	11	25	11	15	30	20	24	21
P6-21-m10	27	24	24	20	11	30	28	18	30	20	27	21
P6-21-m11	18	25	25	13	10	30	26	16	30	23	26	21
P6-21-m12	12	25	26	12	9	30	23	13	29	23	24	19
P6-21-m13	15	21	22	12	10	24	20	16	27	18	21	19
P6-21-m14	18	22	22	12	10	23	20	16	21	11	19	19
P6-21-m15	17	23	24	23	16	32	27	16	32	20	31	20
P6-21-m16	16	23	22	19	15	27	23	16	30	11	30	19
P6-21-m17	19	21	21	25	18	30	24	17	32	13	32	18
P6-21-m18	17	21	23	12	10	22	20	17	24	12	21	19
P6-21-m19	19	28	36	12	10	24	23	17	27	13	20	21
P6-21-m20	16	23	23	14	10	27	20	19	28	16	24	20
P6-21-m21	36	43	36	12	10	21	23	24	24	12	21	20
P6-21-m22	36	40	35	9	9	11	18	26	20	6	13	20
P6-21-m23	14	23	23	18	12	28	21	16	28	15	24	21
P6-21-m24	19	25	25	10	10	26	22	15	30	19	24	21
P6-21-m25	17	24	22	9	9	22	20	14	25	10	21	19
P6-21-m26	13	18	18	17	10	23	20	14	27	13	23	18
P6-21-m27	15	22	21	13	11	12	18	15	21	9	17	19
P6-21-m28	15	22	22	25	10	31	24	17	32	14	27	20
P6-21-m29	17	23	25	15	12	22	20	17	28	12	22	20
P6-21-m30	15	21	21	16	11	21	20	17	24	10	20	19
P6-21-m31	25	27	27	36	28	30	28	37	28	25	26	15
P6-21-m32	27	28	26	36	27	30	30	36	30	25	26	15
P6-21-m33	19	23	23	13	10	25	24	16	26	12	21	19
P6-21-m34	15	20	20	17	10	27	21	18	21	17	25	19
P6-21-m35	18	23	20	19	11	26	22	17	29	21	27	19
P6-21-m36	15	21	20	28	11	26	24	16	25	15	22	20
P6-21-m37	16	24	23	12	13	28	21	15	29	20	23	21
P6-21-m38	25	28	27	36	29	32	28	37	29	26	26	16
P6-21-m39	25	28	27	36	29	20	29	34	31	26	26	15
P6-21-m40	24	28	27	36	28	31	28	36	31	26	25	15

P6-21-m41	18	25	24	11	11	29	21	16	29	21	24	20
P6-21-m42	26	39	33	10	11	23	25	22	24	12	19	20
P6-21-m43	15	21	21	20	10	24	20	18	25	19	22	19
P6-21-m44	18	23	24	21	11	32	24	20	32	19	26	19
P6-21-m45	17	20	22	16	11	28	26	17	29	20	27	20
P6-21-m46	16	21	21	14	10	26	21	16	30	20	25	21
P6-21-m47	16	20	21	13	10	26	22	17	26	22	22	20
P6-21-m48	22	22	22	19	11	26	24	16	30	16	25	21
P6-21-m49	16	22	22	14	10	23	24	18	27	10	24	19
P6-21-m50	16	21	21	20	12	26	23	18	27	17	22	19
P6-21-m51	17	22	21	22	15	32	24	28	32	26	26	19
P6-21-m52	16	21	21	18	11	24	21	18	25	12	22	19
P6-21-m53	18	22	22	18	10	24	26	27	28	15	25	19
P6-21-m54	17	21	22	16	10	22	20	20	24	10	20	18
P6-21-m55	34	39	32	13	10	19	20	21	23	9	19	21
P6-21-m56	17	40	35	10	9	19	22	20	24	8	19	19
P6-21-m57	34	44	37	14	10	27	28	24	28	10	24	19
P6-21-m58	18	25	25	16	9	28	24	15	28	13	20	18
P6-21-m59	19	23	22	12	10	23	20	15	27	10	20	18
P6-21-m60	15	20	19	15	11	23	26	18	24	6	25	20
P6-21-m61	16	23	21	23	12	27	26	15	29	8	27	18
P6-21-m62	18	24	24	15	9	30	24	13	31	13	27	19

AKN amikacin, GME gentamicin, TMN tobramycin, MEM meropenem, IPM imipenem, CAZ ceftazidime, FEP cefepim, CIP ciprofloxacin, ATM aztreonam, PIR piperacillin, TIC ticarcillin, COL colistin. In green or in red, increase or decrease of inhibition zone > or < 2mm, respectively.

Table S2. Strains and plasmids used in the study.

Stains	Description	References
P. aeruginosa		
Reference strain		
PA14	Wild type reference strain, susceptible to antibiotics	[3]
PA14Δ*mexAB*	PA14-derived *mexAB* deleted mutant	[4]
PA14*mexAB*+	Spontaneous MexAB-OprM overproducing mutant *nalB* from PA14, exhibiting a 3-bp deletion in repressor gene *mexR*	[4]
PA14Δ*fliE*	PA14-derived *fliE* deleted mutant	This study
PA14*mutS*::MAR2xT7	PA14-derived *mutS* transposon insertion mutant	[3]
PA14*mutL*::MAR2xT7	PA14-derived *mutL* transposon insertion mutant	[3]
Mutants CNA-R		
CNA-A1, A2, A3	PA14-derived CNA-R mutants obtained by method A after 10 days	This study
CNA-B1, B2, B3	PA14-derived CNA-R mutants obtained by method B after 5 days	This study
CNA-B4, B5, B6, B7	PA14-derived CNA-R mutants obtained by method B after 10 days	This study
B1ΔAB	CNA-B1 with deletion of *mexAB* genes	This study
B3ΔAB	CNA-B3 with deletion of *mexAB* genes	This study
B4ΔAB	CNA-B4 with deletion of *mexAB* genes	This study
B5ΔAB	CNA-B5 with deletion of *mexAB* genes	This study
B6ΔAB	CNA-B6 with deletion of *mexAB* genes	This study
B7ΔAB	CNA-B7 with deletion of *mexAB* genes	This study

Clinical CF-strains		
P6-1	Primo-colonization isolate from patient P6	This study
P6-14	Mid-colonization isolate from patient P6 ($L_{255}Q$ in MutS)	This study
P6-21	Late-colonization isolate from patient P6 ($L_{255}Q$ in MutS)	This study
E. coli		
DH5α	*F- φ80lacZΔM15 Δ(lacZYA-argF) U169 recA1 endA1 hsdR17(rk, mk+) phoA supE44 thi-1 gyrA96 relA1* λ^-	Invitrogen
CC118*λpir*	CC118 lysogenic for phage *λpir*	[5]
HB101	*subE44 subF58 hsdS3*(r_{B}^{-} m_{B}^{-}) *recA13 ara-14 proA2 lacY1 galK2 rpsL20 xyl-5 mtl-1*	[6]
HB101(pRK2013)	Strain HB101 containing helper plasmid pRK2013 for conjugation (*mob1, tra1, colE1,* Kan^R)	[7]
CC118*λpir*(pKNG101)	Strain CC118*λpir* containing suicide vector pKNG101 (*oriR6K, sacB,* Str^R)	[8]

Kan^R, Str^R : selective plasmid markers conferring resistance to kanamycin and streptomycin, respectively.

Table S3: Primers used for gene inactivation and RT-qPCR experiments.

Primer	Sequence (5′→ 3′)	Tm (°C)	References
**Gene inactivation**			
ifliE_2	CGCGAATTCCAGGTGATCAT	60	This study
ifliE_2	GCGCCGCGGAGAGCATTTCCGAAAAGC	60	This study
ifliE_3	GCTCTCCGCGGCGCGAGGATAACTA	60	This study
ifliE_4	GTTGTTCAGGCTCGACACG	60	This study
**RT-qPCR**			
armR-F	CAACAAACCGTCCCGCACCGA	60	[4]
armR-R	TCAGTAGAAGTGCTCGCCGTAGAGG	60	
mexB1	ATCCGCCAGACCATCGCCA	60	[9]
mexB2	CATCACCAGGAACACGAGGAGG	60	
mexY1A	TTACCTCCTCCAGCGGC	60	[10]
mexY1B	GTGAGGCGCGCGTTGTG	60	
mexC1	TGCGGATTTCACCCAGACC	60	[11]
mexC2	GGCCAAGGTGCCTTTCTTCA	60	
mexE4	CCAGGACCAGCACGAACT	60	[11]
mexE5	CGACAACGCCAAGGGCGAGTTCACC	60	
rpsL3	GCAACTATCAACCAGCTGGTG	60	[11]
rpsl5	GCTGTGCTCTTGCAGGTTGTG	60	

Table S4: 18 mutations found in mutant CNA-B1

PA number	Ortholog (PAO1)	*Name*	Mutation	Probable function
				**Transport**
PA14_0167	PA0136		D467G	probable ATP-binding component of ABC transporter
PA14_0241	PA0192		S673G	TonB-dependent receptor protein
PA14_2624	PA2923	*hisJ*	A9T	periplasmic histidine-binding protein HisJ
PA14_5844	PA4503	*dppB*	G114D	dipeptide ABC transporter permease DppB
				**DNA replication/repair**
PA14_0005	PA0004	*gyrB*	Q519R	DNA gyrase, B subunit
**PA14_1750**	**PA3620**	*mutS*	**indel**	DNA mismatch repair protein MutS
				**Transcription or transcriptional regulation**
**PA14_1628**	**PA3721**	*nalC*	**indel**	transcriptional regulator NalC
**PA14_3236**	**PA2497**		**indel**	LysR family transcriptional regulator
PA14_3473	PA2312		G123D	XRE family transcriptional regulator
**PA14_6939**	**PA5255**	*algQ*	**Indel**	anti-RNA polymerase sigma 70 factor
				**Energy and metabolism**
PA14_3882	PA1986	*pqqB*	H246R	pyrroloquinoline quinone biosynthesis protein PqqB
PA14_5221	PA0932	*cysM*	T27A	cysteine synthase B
				**Cell wall integrity**
PA14_2058	PA3364	*amiC*	T233A	aliphatic amidase expression-regulating protein
PA14_6610	PA4999	*waaL*	W282R	Hypothetical protein / O-antigen ligase, WaaL
				**Chemotaxis / stress response**
PA14_2976	PA2652/5	(*tlpQ*)	L561P	chemotaxis transducer
PA14_0372	PA0285		V525A	hypothetical protein (sensory box GGDEF domain-
				**Conjugational transfer**
**PA14_1554**	**No**	*trbL*	**indel**	mating pair formation protein TrbL
				**Unknown function**
PA14_1758	PA3613		R661H	hypothetical protein

In bold, inactivating mutations.

Table S5: 16 mutations found in mutant CNA-B2

PA number	Ortholog	*Name*	Mutation	Probable function
				**Transport**
**PA14_0290**	**PA0235**	***pcaK***	**indel**	4-hydroxybenzoate transporter
				**DNA replication/repair**
PA14_1750	PA3620	*mutS*	Q589R	DNA mismatch repair protein MutS
PA14_5567	PA4284	*recB*	N1172S	exodeoxyribonuclease V, beta subunit
PA14_6301	PA4763	*recN*	T451A	DNA repair protein RecN
				**Transcription or transcriptional regulation**
PA14_0837	PA0652	*vfr*	Y65G	cAMP-regulatory protein / transcriptional regulator
PA14_1406	PA3860		P396L	AMP-binding protein
PA14_5274	PA0893	*argR*	P292S	transcriptional regulator ArgR
				**Translational machineries**
PA14_0875	PA4271	*rplL*	T78A	50S ribosomal protein L7/L12
				**Energy and metabolism**
**PA14_1936**	**PA3460**		**indel**	GNAT family acetyltransferase
PA14_4064	PA1846	*cti*	W441R	cis/trans isomerase
				**Virulence factors**
PA14_1625	PA3725	*lasB*	D365G	elastase LasB
PA14_3279	PA0041		T3644A	hypothetical protein (probable hemagglutinin)
PA14_5043	PA1082	*flgG*	P83L	flagellar basal body rod protein FlgG
				**Chemotaxis / stress response**
PA14_1934	PA3462		G189R	probable sensor/response regulator hybrid
				**Unknown function**
**PA14_3293**	**PA2450**		**V21***	hypothetical protein
PA14_5662	PA4354		D4G	hypothetical protein
PA14_6059	PA4580		A68V	hypothetical protein

In bold, inactivating mutations

Table S6: 26 mutations found in mutant CNA-B3

PA number (PA14)	Ortholog (PAO1)	*Name*	Mutation	Probable function
				**Transport**
PA14_30600	PA2589		A300V	permease
PA14_58440	PA4503	*dppB*	G114D	dipeptide ABC transporter permease DppB
				**DNA replication/repair**
**PA14_17500**	**PA3620**	***mutS***	**indel**	DNA mismatch repair protein MutS
PA14_39300	PA1949	*rbsR*	V320M	ribose operon repressor RbsR
PA14_55610	PA0669	*dnaE2*	G793S	error-prone DNA polymerase
				**Transcription or transcriptional regulation**
**PA14_16280**	**PA3721**	***nalC***	**Indel**	transcriptional regulator NalC
**PA14_49680**	**PA1138**		**Indel**	transcriptional regulator
PA14_11180	PA4074		Q259R	probable transcriptional regulator
				**Energy and metabolism**
PA14_02830	PA0230	*pcaB*	E239G	3-carboxy-cis, cis-muconate cycloisomerase
PA14_15030	PA3792	*leuA*	I464T	2-isopropylmalate synthase
PA14_37370	PA2098		A292V	esterase / deacetylase
PA14_73260	PA5556	*atpA*	D290G	F0F1 ATP synthase, alpha subunit
				**Virulence factors**
PA14_24490	PA3063	*pelB*	S550G	hypothetical protein (pel operon)
**PA14_50160**	**PA1100**	***fliE***	**Q54***	flagellar hook-basal body protein FliE
PA14_58570	6		T735A	outer membrane ferric siderophore receptor
PA14_62350	PA4710	*phuR*	Y295C	heme/hemoglobin uptake outer membrane receptor
				**Chemotaxis / stress response**
PA14_28910	PA2726		F25L	radical activating enzyme
PA14_29760	PA2652/5	*(tlpO)*	L561P	chemotaxis transducer
PA14_11340	PA4061	*ybbN*	F86S	thioredoxin
				**Unknown function**
PA14_21580	PA3283		N142S	hypothetical protein
**PA14_41930**	**PA1749**		**indel**	**hypothetical protein**
PA14_46140	PA1416		V208A	hypothetical protein
**PA14_47120**	**PA1324**		**Indel**	**hypothetical protein**
PA14_53390	PA0841		A304V	hypothetical protein
**PA14_57470**	**PA4422**		**Indel**	**hypothetical protein**
PA14_58070	PA4474		Y365C	hypothetical protein

In bold, inactivating mutations

Table S7: 35 mutations found in mutant CNA-B4

PA number	Ortholog (PAO1)	*Name*	Mutation	Probable function
**Transport**				
PA14_3341	PA2420		V290	Porin
PA14_5844	PA4503	*dppB*	G114	dipeptide ABC transporter permease DppB
PA14_6640	PA5022	*kefA*	F722L	potassium efflux protein KefA
**DNA replication/repair**				
**PA14_1750**	**PA3620**	***mutS***	**indel**	DNA mismatch repair protein MutS
PA14_2648	PA2907	*cobL*	S110P	precorrin-6y-dependent methyltransferase CobL
PA14_3930	PA1949	*rbsR*	V320	ribose operon repressor RbsR
PA14_6838	PA5176	*nudE*	Y45C	ADP-ribose diphosphatase NudE
**Transcription or transcriptional regulation**				
PA14_0878	PA4269	*rpoC*	K1172	DNA-directed RNA polymerase, beta*chain subunit
**PA14_1628**	**PA3721**	***nalC***	**indel**	transcriptional regulator NalC
**PA14_2170**	**PA3271**		**W107**	two-component sensor
**PA14_2370**	**PA3124**		**indel**	LysR family transcriptional regulator
**PA14_4681**	**PA1351**		**indel**	RNA polymerase ECF-subfamily sigma-70 factor
**Translational machineries**				
PA14_1235	PA3280	*yleA/mi*	G200	(dimethylallyl)adenosine tRNA methylthiotransferase
**Energy and metabolism**				
PA14_0758	PA0581	*ygiH*	V120	glycerol-3-phosphate acyltransferase PlsY
PA14_2250	No		F87L	protein-disulfide isomerase
PA14_2486	PA3032	*snr1*	D28G	cytochrome c Snr1
PA14_3348	PA2414	*sndH*	V117	L-sorbosone dehydrogenase
PA14_3420	PA2355		N384	FMNH2-dependent monooxygenase
PA14_7330	PA5559	*atpE*	T23A	F0F1 ATP synthase, C subunit
**Cell wall integrity**				
PA14_4800	3 orthologs		G278	dihydrodipicolinate synthetase
**Virulence factors**				
PA14_0536	PA0411	*pilJ*	T403	twitching motility protein
PA14_1014	PA4161	*fepG*	S177	ferric enterobactin transport protein
**PA14_3363**	**PA2400**	***pvdJ***	**indel**	protein PvdJ synthesis (unknown function)
PA14_5038	PA1085	*flgJ*	Q341	flagellar rod assembly protein/muramidase FlgJ
**Chemotaxis / stress response**				
PA14_2976	PA2652/54	*(tlpQ)*	L561P	chemotaxis transducer
**PA14_4222**	**PA1727**	***mucR***	**indel**	membrane sensor domain-containing protein
PA14_1134	PA4061	*ybbN*	F86S	thioredoxin
**Unknown function**				
**PA14_0206**	**PA0165**		**indel**	hypothetical protein
PA14_0470	PA0360		H297	hypothetical protein
PA14_3080	PA2575		L6P	hypothetical protein
PA14_5538	5 orthologs		G170	hypothetical protein
PA14_6225	PA4703		L61P	hypothetical protein
PA14_6329	PA4788	*phaJ3*	L156P	hypothetical protein
PA14_6646	PA5027		L93S	hypothetical protein
PA14_7058	PA5346	*sadB*	T220	hypothetical protein

In bold, inactivating mutations

Table S8: 24 Mutations found in mutant CNA-B5

PA number (PA14)	Ortholog (PAO1)	*Name*	Mutation	Probable function
				**Transport**
PA14_30600	PA2589		A300V	permease
PA14_58440	PA4503	*dppB*	G114D	dipeptide ABC transporter permease DppB
				**DNA replication/repair**
**PA14_17500**	**PA3620**	***mutS***	**indel**	DNA mismatch repair protein MutS
PA14_39300	PA1949	*rbsR*	V320M	ribose operon repressor RbsR (purines synthesis)
				**Transcription or transcriptional regulation**
PA14_68630	PA5195		Q119R	heat shock protein
**PA14_16280**	**PA3721**	***nalC***	**indel**	transcriptional regulator NalC
				**Translational machineries**
PA14_08820	PA4266	*fusA1*	A225T	elongation factor G
				**Energy and metabolism**
PA14_02830	PA0230	*pcaB*	E239G	3-carboxy-cis, cis-muconate cycloisomerase
PA14_15030	PA3792	*leuA*	I464T	2-isopropylmalate synthase
PA14_63550	PA4809	*fdhE*	T255A	formate dehydrogenase accessory protein FdhE
PA14_73260	PA5556	*atpA*	D290G	F0F1 ATP synthase, alpha subunit
				**Cell wall integrity**
PA14_73040	PA5538	*amiA*	R62W	N-acetylmuramoyl-L-alanine amidase
				**Virulence factors**
PA14_24490	PA3063	*pelB*	S550G	hypothetical protein (operon pel)
**PA14_50160**	**PA1100**	***fliE***	**Q54***	flagellar hook-basal body protein FliE
PA14_62350	PA4710	*phuR*	Y295C	heme/hemoglobin uptake outer membrane receptor PhuR
				**Chemotaxis / stress response**
PA14_29760	PA2652/54	*(tlpQ)*	L561P	chemotaxis transducer
PA14_11340	PA4061	*ybbN*	F86S	thioredoxin
				**Unknown function**
PA14_21580	PA3283		N142S	Hypothetical protein

PA14_27560	PA2823		R271C	hypothetical protein
PA14_46140	PA1416		V208A	hypothetical protein
PA14_53390	PA0841		A304V	hypothetical protein
**PA14_57470**	**PA4422**		**Indel**	hypothetical protein
PA14_57680	PA4440		V146A	hypothetical protein
PA14_58070	PA4474		Y365C	hypothetical protein

In bold, inactivating mutations

Table S9: 35 mutations found in mutant CNA-B6

PA number	Ortholog (PAO1)	*Name*	Mutation	Probable function
				**Transport**
PA14_10200	PA4156	*fvbA*	T59A	TonB-dependent receptor protein
PA14_45060	PA1497		G253S	transporter
PA14_58440	PA4503	*dppB*	G114D	dipeptide ABC transporter permease DppB
PA14_66400	PA5022	*kefA*	F722L	potassium efflux protein KefA
PA14_68060	PA5152		H256R	ABC transporter ATP-binding protein
				**DNA replication/repair**
PA14_15310	PA3770	*guaB*	P154S	inosine 5′-monophosphate dehydrogenase
**PA14_17500**	**PA3620**	***mutS***	**indel**	DNA mismatch repair protein MutS
PA14_39300	PA1949	*rbsR*	V320M	ribose operon repressor RbsR
**PA14_51650**	**No ortholog**		**Indel**	integrase
PA14_68380	PA5176	*nudE*	Y45C	ADP-ribose diphosphatase NudE
PA14_72490	PA5493	*polA*	P609L	DNA polymerase I
				**Transcription or transcriptional regulation**
PA14_08780	PA4269	*rpoC*	K1172E	DNA-directed RNA polymerase, beta*chain subunit
**PA14_16280**	**PA3721**	***nalC***	**indel**	transcriptional regulator NalC
PA14_16380	PA3711		V272A	LysR family transcriptional regulator
				**Translational machineries**
PA14-00180	PA0017		A253V	hypothetical protein (tRNA and rRNA cytosine C5-methylases)
				**Energy and metabolism**
PA14_10260	PA4150		A219T	dehydrogenase E1 component
PA14_11130	PA4079		V4A	short chain dehydrogenase / NADPH-dependant carbonyl reductase
PA14_38200	PA2035		V442A	thiamine pyrophosphate protein
PA14_51220	6 orthologs	*wapB*	Y192C	glycosyl transferase family protein – 1,2-glucosyltransferase WapB
PA14_53380	PA0842		G399E	glycosyl transferase family protein
				**Cell wall integrity**

PA14_56450	3 orthologs		P229L	amidase
				**Virulence factors**
PA14_05360	PA0411	*pilJ*	T403A	twitching motility protein
PA14_42660	PA1690	*pscU*	N263S	translocation protein in type III secretion
PA14_50380	PA1085	*flgJ*	Q341R	flagellar rod assembly protein/muramidase FlgJ
				**Chemotaxis / stress response**
**PA14_21700**	**PA3271**	***mxtR***	**W1071***	two-component sensor
PA14_29760	PA2652/54	*(tlpQ)*	L561P	chemotaxis transducer
PA14_11340	PA4061	*ybbN*	F86S	thioredoxin
				**Unknown function**
**PA14_00990**	**PA0082**	***tssA1***	**indel**	hypothetical protein
**PA14_02060**	**PA0165**		**Indel**	hypothetical protein
PA14_27350	PA2842		L136P	hypothetical protein
PA14_55380	5 orthologs		G170D	hypothetical protein
PA14_57650	PA4438		T256A	hypothetical protein
PA14_60070	PA1939		S85P	hypothetical protein
PA14_62250	PA4703		L61P	hypothetical protein
**PA14_73140**	**PA5546**	***dppB***	**Indel**	hypothetical protein (conserved)

In bold, inactivating mutations

Table S10: 39 mutations found in mutant CNA-B7

PA number (PA14)	Ortholog (PAO1)	*Name*	Mutation	Probable function
				**Transport**
PA14_06340	PA0487	*modR/modE*	I171T	probable molybdenum transport regulator
PA14_13170	PA3920	*copA1*	E150G	metal transporting P-type ATPase
PA14_15475	No	*merT*	L101P	mercuric transport protein MerT
PA14_18275	PA3560	*fruA*	K236R	phosphotransferase system, transporter fructose-specific
PA14_21620	PA3279	*oprP*	V288A	phosphate-specific outer membrane porin OprP
**PA14_50550**	**PA1071**	***livG/braF***	**Indel**	leucine/isoleucine/valine transporter ATP-binding subunit/branched-chain amino acid transport protein
PA14_58440	PA4503	*dppB*	G114D	dipeptide ABC transporter permease DppB
				**DNA replication/repair**
PA14_00010	PA0001	*dnaA*	A338V	chromosomal replication initiator protein
**PA14_17500**	**PA3620**	***mutS***	**Indel**	DNA mismatch repair protein MutS
PA14_39300	PA1949	*rbsR*	V320M	ribose operon repressor RbsR
				**Translational machineries**
PA14_70420	PA5334	*rph*	T71I	ribonuclease PH
				**Energy and metabolism**
PA14_02830	PA0230	*pcaB*	E239G	3-carboxy-cis, cis-muconate cycloisomerase
PA14_35270	PA2266		V338A	cytochrome c precursor
PA14_35880	No		P321L	gamma-aminobutyraldehyde dehydrogenase
PA14_37470	PA2090		V125A	flavin-dependent oxidoreductase
**PA14_50010**			**Indel**	dehydrogenase
PA14_53220	PA0854	*fumC2*	G231S	fumarate hydratase
				**Transcription or transcriptional regulation**
**PA14_16280**	**PA3721**	***nalC***	**Indel**	transcriptional regulator NalC
				**Cell wall integrity**
PA14_11845	PA4020	*mpl*	P237S	UDP-N-acetylmuramate: L-alanyl-gamma-D-glutamyl-meso-diaminopimelate ligase
				**Virulence factors**
**PA14_50160**	**PA1100**	***fliE***	**Q54***	flagellar hook-basal body protein FliE
PA14_63960	6	*cntO*	L659P	cntO (TonB-dependent outer-membrane protein)
				**Chemotaxis / stress response**
PA14_11340	PA4061	*ybbN*	F86S	thioredoxin
PA14_21700	PA3271	*mxtR*	L1068P	two-component sensor
PA14_29760	PA2652/54	*(tlpQ)*	L561P	chemotaxis transducer
**PA14_36020**	**No**		**Indel**	paraquat-inducible protein B
PA14_58650	PA4520		T282A	chemotaxis transducer
PA14_72380	PA5483	*algB*	R307H	two-component response regulator AlgB
				**Unknown function**
PA14_03470	PA0267		L234F	hypothetical protein
PA14_13130	PA3923		P281L	hypothetical protein
PA14_15600	No		S4P	hypothetical protein
PA14_21580	PA3283		N142S	hypothetical protein
**PA14_21630**	**PA3278**		**Indel**	hypothetical protein
PA14_21710	PA3270		H171R	hypothetical protein
PA14_44100	PA1578		N234S	hypothetical protein
**PA14_49030**	**No**		**Indel**	hypothetical protein
PA14_50070	PA1106		Y204C	hypothetical protein
PA14_55380	5		Q450R	hypothetical protein
PA14_58250	PA4489	*magD*	V898A	hypothetical protein
PA14_59480	No	*modR/modE*	V75A	hypothetical protein

In bold, inactivating mutations

References

1. Robert, X.; Gouet, P. Deciphering key features in protein structures with the new ENDscript server. *Nucleic Acids Res* **2014**, *42*, W320-324, doi:10.1093/nar/gku316.
2. Sievers, F.; Wilm, A.; Dineen, D.; Gibson, T.J.; Karplus, K.; Li, W.; Lopez, R.; McWilliam, H.; Remmert, M.; Soding, J.; et al. Fast, scalable generation of high-quality protein multiple sequence alignments using Clustal Omega. *Mol Syst Biol* **2011**, *7*, 539, doi:10.1038/msb.2011.75.
3. Liberati, N.T.; Urbach, J.M.; Miyata, S.; Lee, D.G.; Drenkard, E.; Wu, G.; Villanueva, J.; Wei, T.; Ausubel, F.M. An ordered, nonredundant library of *Pseudomonas aeruginosa* strain PA14 transposon insertion mutants. *Proc Natl Acad Sci U S A* **2006**, *103*, 2833-2838, doi:0511100103 [pii] 10.1073/pnas.0511100103.
4. Tetard, A.; Zedet, A.; Girard, C.; Plésiat, P.; Llanes, C. Cinnamaldehyde induces expression of efflux pumps and multidrug resistance in *Pseudomonas aeruginosa*. *Antimicrob Agents Chemother* **2019**, *63*, doi:10.1128/AAC.01081-19.
5. Herrero, M.; de Lorenzo, V.; Timmis, K.N. Transposon vectors containing non-antibiotic resistance selection markers for cloning and stable chromosomal insertion of foreign genes in Gram-negative bacteria. *J Bacteriol* **1990**, *172*, 6557-6567.
6. Lacks, S.; Greenberg, B. Complementary specificity of restriction endonucleases of *Diplococcus pneumoniae* with respect to DNA methylation. *J Mol Biol* **1977**, *114*, 153-168.
7. Ditta, G.; Stanfield, S.; Corbin, D.; Helinski, D.R. Broad host range DNA cloning system for gram-negative bacteria: construction of a gene bank of *Rhizobium meliloti*. *Proc Natl Acad Sci U S A* **1980**, *77*, 7347-7351.
8. Kaniga, K.; Delor, I.; Cornelis, G.R. A wide-host-range suicide vector for improving reverse genetics in Gram-negative bacteria: inactivation of the *blaA* gene of *Yersinia enterocolitica*. *Gene* **1991**, *109*, 137-141, doi:0378-1119(91)90599-7 [pii].
9. Hocquet, D.; Nordmann, P.; El Garch, F.; Cabanne, L.; Plésiat, P. Involvement of the MexXY-OprM efflux system in emergence of cefepime resistance in clinical strains of *Pseudomonas aeruginosa*. *Antimicrob Agents Chemother* **2006**, *50*, 1347-1351.
10. Jeannot, K.; Sobel, M.L.; El Garch, F.; Poole, K.; Plésiat, P. Induction of the MexXY efflux pump in *Pseudomonas aeruginosa* is dependent on drug-ribosome interaction. *J Bacteriol* **2005**, *187*, 5341-5346.
11. Dumas, J.L.; van Delden, C.; Perron, K.; Köhler, T. Analysis of antibiotic resistance gene expression in *Pseudomonas aeruginosa* by quantitative real-time-PCR. *FEMS Microbiol Lett* **2006**, *254*, 217-225.
